# Myeloid-specific knockout of Notch-1 inhibits MyD88- and TRIF-mediated TLR signaling pathways by regulating oxidative stress-SHP2 axis, thus restraining aneurysm progression

**DOI:** 10.18632/aging.205392

**Published:** 2024-01-26

**Authors:** Yu Li, Ailin Guo, Jianlei Liu, Lijuan Tang, Lide Su, Zonghong Liu

**Affiliations:** 1 Department of Cardiovascular Surgery, Xiang’an Hospital of Xiamen University, School of Medicine, Xiamen University, Xiamen 361102, China; 2 Institute of Prevention and Control of Non-communicable Chronic Diseases, Hebei Province Center for Disease Prevention and Control, Shijiazhuang 050021, China

**Keywords:** abdominal aortic aneurysm, Notch1, macrophages, oxidative stress, SHP2, MyD88/TRIF/NF-κB signaling pathway

## Abstract

Objective: Notch-1 is a signal regulatory protein with extensive effects in myeloid cells, but its role in aneurysms remains to be fully clarified. In this study, therefore, the aneurysm mouse model with myeloid-specific knockout of Notch-1 was established to observe the role of Notch-1 in aneurysm progression.

Methods and Results: The effect of Notch-1 was assessed by pathological staining and Western blotting. It was found that after myeloid-specific knockout of Notch-1 in the aneurysm mouse model, the area of aneurysms and the macrophage infiltration were significantly reduced, the damage to arterial elastic plates was significantly relieved, and the oxidative stress level significantly declined. The results of Western blotting showed that after myeloid-specific knockout of Notch-1, the levels of oxidative stress-related proteins p22 and p47 in aneurysm tissues significantly declined, accompanied by a significant increase in the protein level of Src homology 2 domain-containing tyrosine phosphatase-2 (SHP2). In addition, the levels of phosphorylated myeloid differential protein-88 (MyD88), TIR domain-containing adaptor-inducing interferon-β (TRIF) and nuclear factor-κB (NF-κB), and inflammatory cytokines interferon-γ (IFN-γ), interleukin-1β (IL-1β) and tumor necrosis factor-α (TNF-α) also significantly decreased after myeloid-specific knockout of Notch-1. Following myeloid-specific knockout of Notch-1, the phagocytic capacity of macrophages was enhanced by promoting the SHP2 signaling pathway.

Conclusion: Notch-1 in monocytes/macrophages can activate the Toll-like receptor (TLR)-mediated inflammatory and stress responses by activating oxidative stress and inhibiting the SHP2 protein expression, thus facilitating aneurysm progression.

## INTRODUCTION

Abdominal aortic aneurysm (AAA), a common and potentially life-threatening degenerative vascular disease in the elderly [1], is defined as focal dilatation of the abdominal aorta beyond 50% of the normal diameter, which is characterized by destruction of elastin and collagen in the media and adventitia, loss of smooth muscle cells, thinning of the medial wall, infiltration of lymphocytes and macrophages, and neovascularization [2]. At present, the pathogenesis of AAA remains unclear, thus hindering the development of effective therapeutic strategies, so that surgical intervention becomes the only therapeutic option. It has been verified that inflammatory cells are accumulated in the adventitia by recruiting circulating monocytes or proliferating macrophages in AAA [3]. Innate and acquired immune-mediated inflammatory responses are an important player in the formation of aneurysms, which have attracted increasingly more attention from basic and clinical researchers [4].

Both Notch-1 and oxidative stress are reliable biomarkers for the diagnosis of AAA [5]. Notch-1 is a signal regulatory protein with extensive effects in myeloid cells, and the Notch-1 signaling pathway, a conserved intercellular signaling pathway, has been widely recognized as an important role in the development and functional evolution of bone marrow-derived cells [6]. Pattern recognition receptors (PRRs) on the cell surface and their signal transduction are of high importance in the activation and inflammatory response of these myeloid cells [7]. The intracellular signal transduction of Toll-like receptors (TLRs), a member of the PRR family, is achieved mainly in a myeloid differential protein-88 (MyD88)-dependent and -independent way, namely MyD88 and TIR domain-containing adaptor-inducing interferon-β (TRIF) pathway [8], which are both negatively regulated by Src homology 2 domain-containing tyrosine phosphatase-2 (SHP2) directly [9, 10]. Mechanically, SHP2 directly interacts with MyD88 via its tyrosine 257, and it also specifically negatively regulates TRIF-mediated gene expression in the TLR signaling pathway. Therefore, the expression of SHP2 can affect the TLR-induced inflammatory responses. Oxidative stress can directly cause the degradation of SHP2, and then the degradation of SHP2 causes oxidative stress by activating ANT1-dependent mitochondrial homeostasis, thereby forming a vicious cycle. The TLR signaling pathway can mediate MyD88/TRIF-dependent signal transduction, involving such signaling molecules as IRAKs, TRAF6, TAK1 and downstream IKKs and NF-κB [11]. Ultimately, the inflammatory response in monocytes/macrophages is induced by the cascade reaction of these molecules, and inflammatory factors such as interferon-γ (IFN-γ), interleukin-1β (IL-1β) and tumor necrosis factor-α (TNF-α) are released, allowing macrophages and other inflammatory immune cells in aneurysms to exert their biological effects [11]. It has been found that MyD88 knockout and targeted knockout of TLR4-encoding genes in hypercholesterolemia mouse models can both inhibit the formation of aneurysms [12]. Therefore, blocking the TLR signal transduction in myeloid cells, especially macrophages, is an important means for inhibiting aneurysm progression. In this study, the mouse model with myeloid-specific knockout of Notch-1 was established by hybridization of ApoE^−/−^/Lyz2-Cre^+/−^ mice and ApoE^−/−^/Notch-1^flox/flox^ mice, and then the regulatory effect of Notch-1 on TLR-mediated inflammation and its molecular mechanism were explored. At the same time, the influence of Notch-1 on aneurysm formation was clarified, thereby providing new molecular mechanisms and potential intervention targets for aneurysm formation and progression.

## MATERIALS AND METHODS

### Construction of Notch-1-knockout mice

ApoE^−/−^/Lyz2-Cre^+/−^ mice and ApoE^−/−^/Notch-1^flox/flox^ mice were constructed from ApoE^−/−^ mice purchased from Xiang'an Hospital of Xiamen University, specifically as follows: Transgenic vectors containing the Lyz2-Cre gene were constructed, the fertilized eggs were implanted into the uterus of the female mouse, and then the Lyz2-Cre^+/−^ mice were screened. Based on the Notch-1 gene sequence, specific primers were designed to insert two loxP sites, constructing transgenic vectors. Transgenic vectors should also include elements such as selective marker genes, which were used for *in vitro* DNA recombination. Then recombinant DNA was transfected into mouse embryonic stem cells for hybridization and recombination, and Notch-1^flox/flox^ embryonic stem cells were injected into normally developing mouse embryos. The embryos were implanted into the uterus of the female mouse. Finally, Notch-1^flox/flox^ mice were screened. All mice were housed in SPF rooms with a 12 h/12 h light-dark cycle, and were given adequate diet and water.

### Establishment of ApoE^−/−^ chimeric mouse model (ApoE-KO/Notch-1^MAC-KO^)

18 Male 12-weeks old ApoE^−/−^ mice were given gentamicin sulfate (320,000 U/L) in the drinking water from 1 week before transplantation. On the day of transplantation, ApoE^−/−^ mice were subjected to total body irradiation by X-ray at a total dose of 10 Gy (0.8 Gy/min, twice at an interval of 4 h). The mice were divided into the ApoE-KO/Notch-1^WT^ group and the ApoE-KO/Notch-1^MAC-KO^ group.9 mice per group. Bone marrow cells of KO mice were prepared into mononuclear cell (MNC) suspension with serum-containing RPMI1640 medium, and the cell concentration was adjusted to 5 × 10^7^ MNCs/mL. Following irradiation for 4–6 h, 5 × 10^6^ MNCs were injected via the caudal vein for hematopoietic reconstitution. 6 weeks later, the successfully chimeric mice were pumped with angiotensin II at 1000 ng/kg/min to induce aneurysm formation (AAA group), while the remaining mice were pumped with normal saline (CTL group).

### Sampling

All mice were sacrificed by decapitation 28 d later, and the aortic tissue and blood were promptly harvested for further analysis. The tissues of four mice in each group were fixed with 4% paraformaldehyde followed by histological observation, while those of the remaining mice in each group were stored at −80°C for later molecular assays. During sampling, the adipose tissue of vascular adventitia was peeled off, the morphology of aorta was observed, and the maximum diameter of vessel was measured.

### Histological observation

After drying, the sections were mounted with gum, and the change in tissue morphology was observed under an optical microscope. Following dehydration with gradient ethanol, the sections were stained with Weigert's iron hematoxylin solution, water-washed and differentiated with acidic ethanol differentiation solution. After washing, the sections were added with Masson staining solution, washed again, stained with Ponceau acid fuchsin staining solution, washed with phosphomolybdic acid solution, added with aniline blue dye, and washed with weak acid solution. Finally, the sections were dehydrated with gradient ethanol, transparentized with xylene and mounted with neutral gum, followed by microscopic observation.

### Immunofluorescence and immunohistochemical staining

After complete drying, the 4 μm-thick paraffin sections were deparaffinized with xylene, rehydrated with gradient ethanol, and incubated with proteinase K solution at room temperature for 20 min, followed by incubation with primary antibodies at 4°C overnight and with corresponding fluorescence-based HRP-labeled secondary antibodies (1:500) at room temperature for 1 h. After washing with PBS, the sections were stained with DAPI or hematoxylin. Finally, the macrophage infiltration and the distribution of Notch-1 in aortic aneurysms were observed under a fluorescence or bright-field microscope, and the fluorescence was excited at the corresponding wavelength and merged during multiple staining. The results were photographed and recorded.

### Western blotting

The protein was extracted from tissues of ApoE^−/−^ mice using RIPA lysis buffer and PMSF. Specifically, the tissues were lysed with lysis buffer containing the protease inhibitor PMSF (PMSF: RIPA=1:100) on ice for 30 min to extract the protein. Then the protein was separated by SDS-PAGE, transferred onto a PVDF membrane, and incubated with antibodies against iNOS, IL-10, Arginase-1, GAPDH, p22, p47, p-MyD88, p-TRIF, p-NF-κB, IFN-γ, IL-1β and TNF-α (1:1000) at 4°C overnight. Finally, the membrane was incubated again with HRP-labeled goat anti-rabbit or goat anti-mouse IgG secondary antibodies at room temperature for 2 h, exposed using the Ultra High Sensitivity ECL Kit and FluorChem Q, and quantified by AlphaView software.

### Extraction of bone marrow-derived macrophages and phagocytosis assay with fluorescent microspheres

The mice were sacrificed and thoroughly disinfected with 75% alcohol. The tibia and femur of mice were isolated under sterile conditions, placed in a cell culture dish containing 75% alcohol, washed with PBS 2–3 times, and transferred to a cell culture dish containing complete medium (1% penicillin-streptomycin + 10% fetal bovine serum + DMEM basal medium). Then both ends of the tibia and femur were cut with ophthalmic scissors, and rinsed with complete medium pipetted with a 1 mL syringe to harvest the bone marrow cells from one end of the bone into a 50 mL sterile centrifuge tube, and the operation was repeated several times until the bones became white. After 5-fold red blood cell lysis buffer was added to the centrifuge tube, the cells were pipetted repeatedly, and left to stand for 15 min, followed by centrifugation at 1000 rpm. After the supernatant was discarded, the cells were resuspended with an appropriate amount of DMEM and filtered through a 200-mesh strainer, followed by centrifugation at 1,000 rpm for 10 min, and the supernatant was discarded. The above operation was repeated twice. Finally, the supernatant was discarded, and the cells were resuspended with DMEM containing 10 ng/mL M-CSF to induce differentiation into macrophages. The resulting macrophages were then treated with the SHP2 inhibitor PHPS1.

The macrophages were incubated with 10 μm fluorescent silver YG microspheres, and pre-incubated in 2.1% BSA (Polysciences; 4.55 × 10^6^ beads/mL) for 24 h. Then they were washed fully with PBS, fixed with 2% paraformaldehyde in PBS, counterstained with 4’,6-diamidino-2-phenylindole and 1,1’-octacosyl-3,3,3’,3’-tetramethylindoline according to the manufacturer's instructions, and mounted on slides with Vectashield mounting media.

### Statistical analysis

Data were analyzed using GraphPad Prism 9.0 and described by mean ± standard deviation. The difference was compared by the Student’s *t*-test between two groups, and by one-way analysis of variance among groups. ^*^P < 0.05 was considered statistically significant. All assays were repeated three times independently.

### Data availability

Our data can be obtained by contacting the corresponding author.

## RESULTS

### Notch-1 in macrophage was implicated in AAA

**Figure 1:**
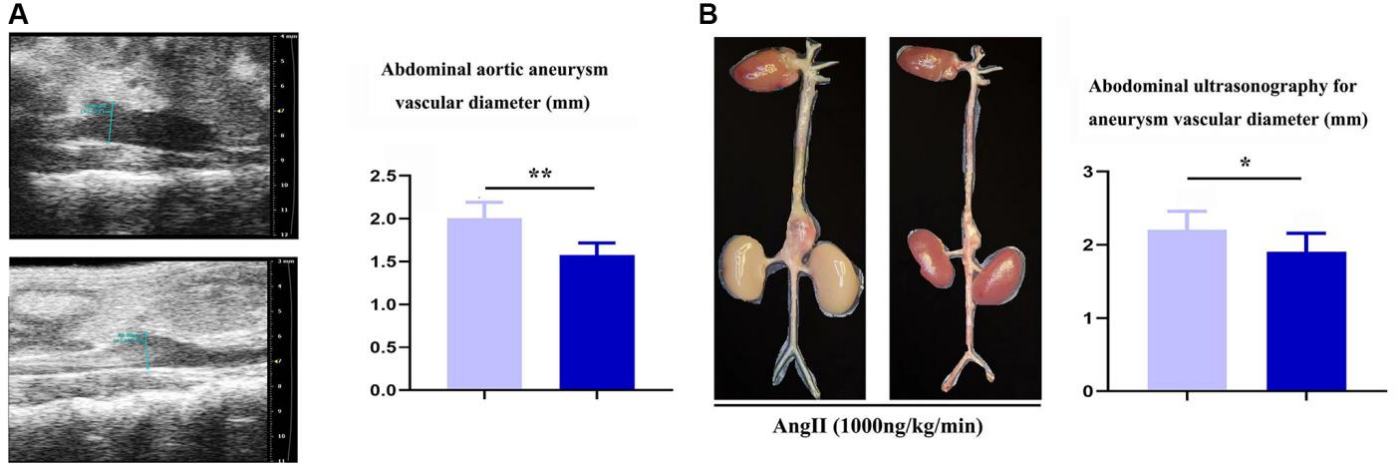
**Specific deficiency of notch-1 in macrophage suppressed the progression of AAA.** (**A**) representative aortic images with identical magnification and quantitative analysis; (**B**) two-dimensional B-mode high-resolution ultrasonography and quantitative analysis. *N* = 9, ^*^P < 0.05, ^**^P < 0.01, apoE-KO/Notch-1^MAC-KO^ group vs. apoE-KO/Notch-1^WT^ group.

To assess the role of Notch-1 in macrophages in the development of AAA, aneurysm mouse models were established by bone marrow transplantation, and the mice were divided into the ApoE-KO/Notch-1^WT^ group and ApoE-KO/Notch-1^MAC-KO^ group. As observed by two-dimensional B-mode high-resolution ultrasonography, ApoE-KO/Notch-1^MAC-KO^ group exhibited a significantly smaller lumen diameter than the ApoE-KO/Notch-1^WT^ group (*P* < 0.01), which was also verified by the quantitative analysis. The lumen of the artery refers to the lumen in which blood flows through the artery, and the space inside the artery. In AAA, the lumen of the artery refers to the space surrounded by the dilated aortic wall. These results implied that knockout of Notch-1 in macrophages can reduce the aortic dilation and the lumen diameters in ApoE-KO/Notch-1^WT^ mice. Besides, these mice were infused with angiotensin II to induce AAA. Abdominal aorta sections (including the aneurysm) were surgically harvested, and representative aortic images are shown in Figure 1. It was found that the aortic diameters increased, suggesting that the AAA mouse models were established successfully. Furthermore, smaller aortic diameters were found in the CTL group than the AAA group (*P* < 0.05, *P* = 0.0211).

**Figure 2:**
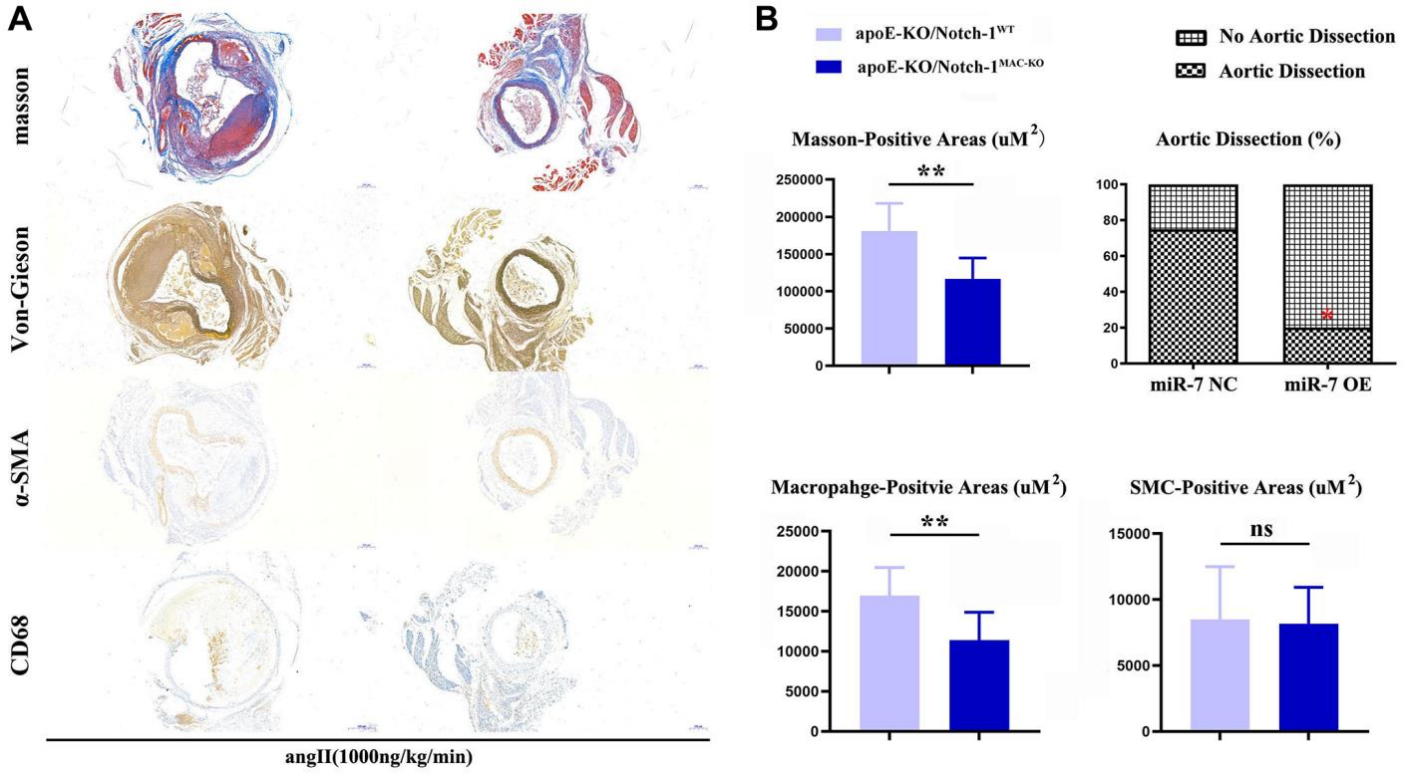
**Notch-1-cKO in macrophages inhibited the elastic lamina degradation and macrophages infiltration in AAA.** (**A**) representative image for Masson’s staining, α-SMA staining, CD68 immunostaining, and Verhoeff-van Gieson staining; (**B**) statistical data for AAA areas, macrophages, smooth muscle cells as well as ratio of aortic dissection. *N* = 9, ^*^P < 0.05, ^**^P < 0.01, apoE-KO/Notch-1^MAC-KO^ group vs. apoE-KO/Notch-1^WT^ group.

The results of Masson staining, EVG staining and immunohistochemical staining showed that the macrophage-positive area and the area of aneurysms in the ApoE-KO/Notch-1^MAC-KO^ group significantly decreased compared with the ApoE-KO/Notch-1^WT^ group (*P* < 0.01), while the SMC-positive area was not significantly different between the two groups (*P* > 0.05, *P* = 0.8394), indicating that macrophages play a major role in the regulation of AAA (Figure 2).

### Effect of Notch-1 on macrophage polarization

**Figure 3:**
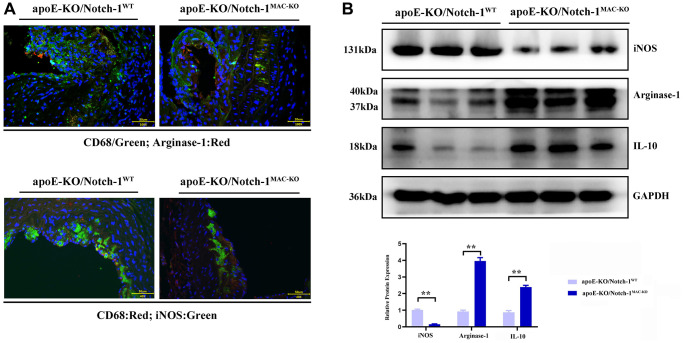
**Notch-1 cKO in macrophages promote the M2 polarization in AAA.** (**A**) Immunofluorescence staining results showed the Notch-1 deficiency in macrophages inhibited the expression of iNOS (M1 marker) and increased the expression of arginase-1 (M2 marker) and statistical data; (**B**) macrophages’ deficiency of Notch-1 decreased the expression of iNOS and increased the expression of arginase-1 and IL-10 in AAA tested by western blot and the quantitative analysis. *N* = 6, ^*^P < 0.05, ^**^P < 0.01, apoE-KO/Notch-1^MAC-KO^ group vs. apoE-KO/Notch-1^WT^ group.

The results of Western blotting revealed that knockout of Notch-1 decreased the expression of iNOS (an M1 macrophage marker) (*P* < 0.01), but increased the expressions of IL-10 and Arginase-1 (M2 macrophage markers) (*P* < 0.01). It can be seen that Notch-1 may promote M1 macrophage polarization, while knockout of Notch-1 promotes M2 macrophage polarization. Besides, the immunofluorescence results showed that the number of iNOS-expressing macrophages in Notch-1-knockout mice decreased after M1 polarization, i.e., M1 polarization was impaired after the knockout of Notch-1. In contrast, the number of Arginase-1-expressing macrophages in Notch-1-knockout mice increased after M2 polarization (Figure 3).

### Myeloid-specific knockout of Notch-1 regulated oxidative stress and SHP2 protein expression

**Figure 4:**
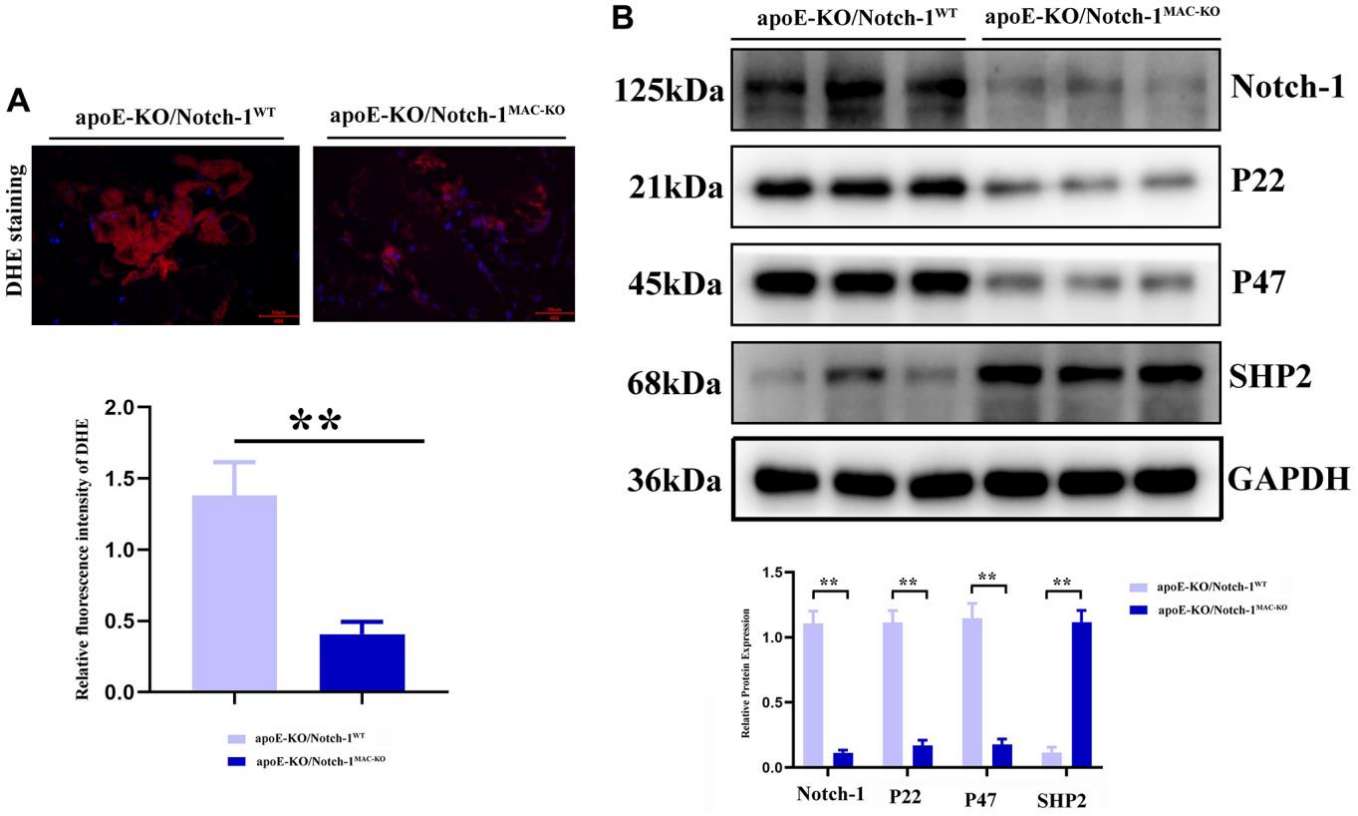
**Notch-1 cKO in macrophages increased the SHP2 and suppressed the ROS in AAA.** (**A**) DHE staining results showed the macrophages’ deficiency of Notch-1 suppressed the ROS production and its statistical data. *N* = 9; (**B**) macrophages’ deficiency of Notch-1 suppressed the expression of Notch-1, p22, p47 and consistent with increased expression of SHP2 in AAA tested by western blot. ^*^P < 0.05, ^**^P < 0.01, apoE-KO/Notch-1^MAC-KO^ group vs. apoE-KO/Notch-1^WT^ group.

Oxidative stress and inflammatory response are typical characteristics of aneurysms, in which SHP2 plays a variety of roles in different cells, different environments and different stages. Therefore, the role of SHP2 in the inflammatory response or inflammatory environment was explored. It was found that compared with those in the CTL group, the protein expressions of p22 and p47 significantly decreased in the Notch-1^MAC-KO^ mice, accompanied by an increase in the protein expression of SHP2 (*P* < 0.01). The above findings suggest that Notch-1 can exert a pro-inflammatory effect through the oxidative stress-SHP2 axis (Figure 4).

### Myeloid-specific knockout of Notch-1 suppressed p-MyD88/TRIF pathway-mediated inflammatory responses

**Figure 5:**
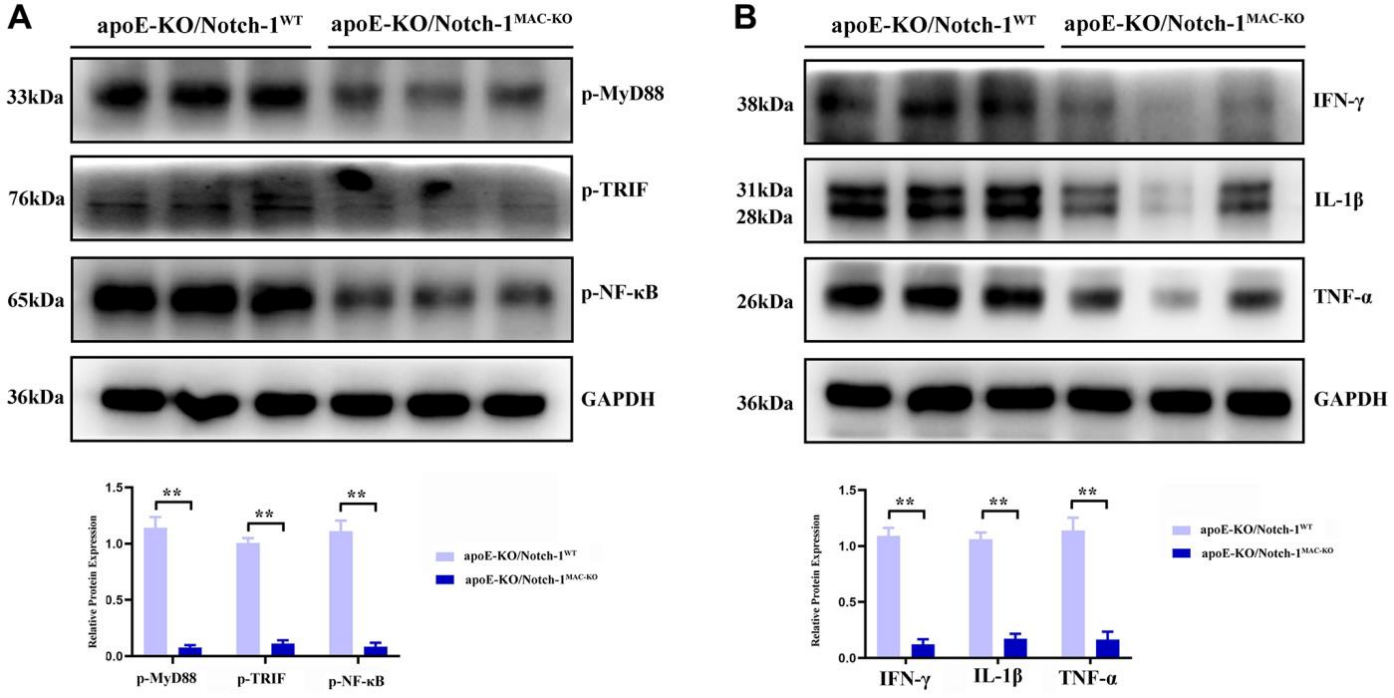
**Notch-1 deficiency inhibited the activation of MyD88/TRIF/NF-κB signals.** (**A**) Macrophages’ deficiency of Notch-1 inhibited the phosphorylated-MyD88, TRIF, and NF-κB and there statistical data; (**B**) Notch-1 cKO decreased the expression of IFN-γ, IL-1β, TNF-α expression in AAA tested by western blot. *N* = 6, ^*^P < 0.05, ^**^P < 0.01, apoE-KO/Notch-1^MAC-KO^ group vs. apoE-KO/Notch-1^WT^ group.

MyD88/TRIF pathway, which activates NF-κB accompanied by an excessive expression of inflammatory factors, is the major and key pathway accelerating aneurysm progression. Then more inflammatory cells, especially M1 macrophages, accumulate toward the aneurysm, thus accelerating aneurysm progression [13–16]. The results of Western blotting showed that after treatment with angiotensin II, the MyD88/TRIF/NF-κB signaling pathway was activated, thus inducing an inflammatory response in the artery. In Notch-1^MAC-KO^ mice, the phosphorylation levels of the MyD88/TRIF/NF-κB signaling pathway-related proteins were significantly inhibited, accompanied by significant decreases in the protein expressions of inflammatory factors IFN-γ, IL-1β and TNF-α (*P* < 0.01) (Figure 5).

### Myeloid-specific knockout of Notch-1 enhanced phagocytic capacity of macrophages by promoting the SHP2 signaling pathway

**Figure 6:**
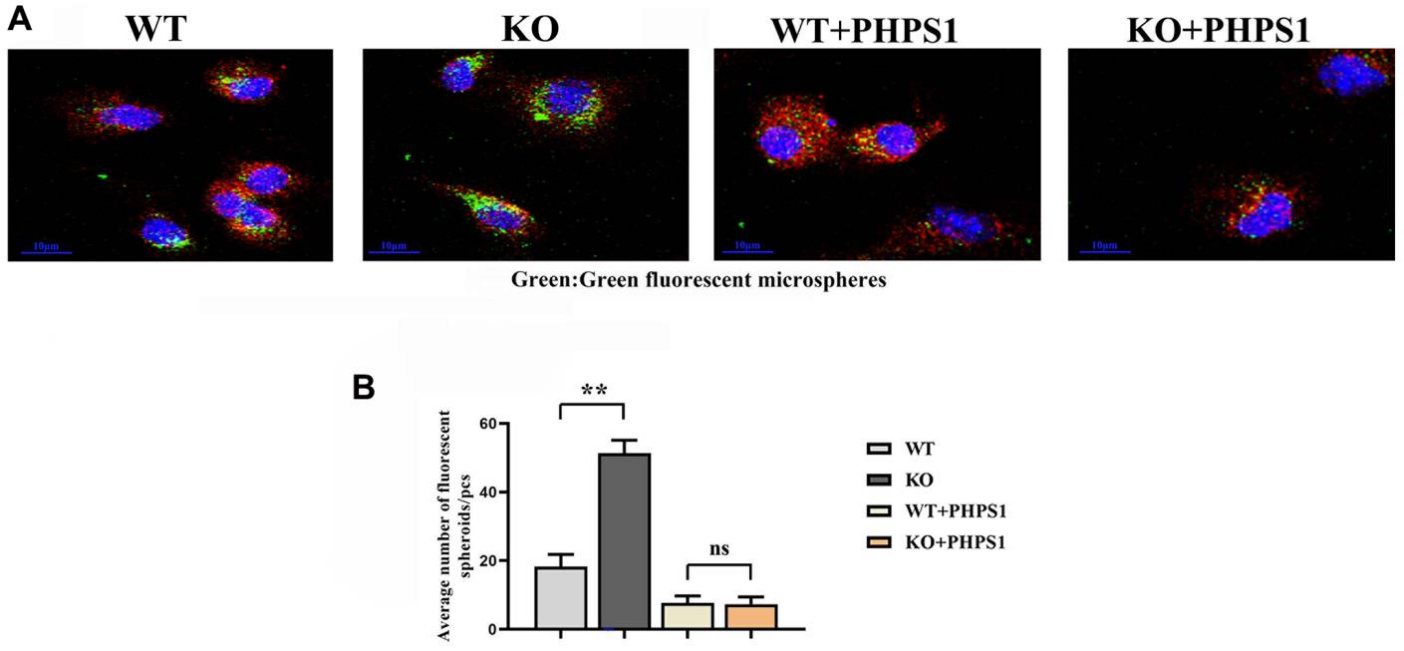
**Monocytes/macrophages-specific knockout of Notch1 promotes phagocytic capacity by promoting the SHP2 signaling pathway.** (**A**) Immunofluorescent spheroid phagocytosis experimental results; (**B**) Macrophage engulfs fluorescent spheroid count statistics. *N* = 3, ^**^P < 0.01, WT group vs. KO group, WT+PHPS1 group vs. KO+PHPS1 group.

To verify the effect of myeloid-specific knockout of Notch-1 on the phagocytic capacity of macrophages, the phagocytosis assay with fluorescent microspheres was designed. The results showed that the number of fluorescent microspheres phagocytosed in the ApoE-KO/Notch-1^MAC-KO^ group was significantly larger than that in the ApoE-KO/Notch-1^WT^ group. However, after the addition of PHPS1, the number of fluorescent microspheres phagocytosed decreased significantly in both groups, without significant differences (Figure 6).

## DISCUSSION

Inflammatory cell infiltration in the arterial wall is a typical pathological change of AAA. The Notch signaling pathway plays an important role in atherosclerosis and arterial wall inflammation, and Notch-1 receptor is one of its important components [17]. Activated Notch-1 can induce M1 and M2 macrophage polarization, and cause AAA through macrophage-mediated inflammatory responses [18]. As shown in previous studies, TLRs are the major and important regulator for macrophage polarization during the onset of aneurysms [19]. The feasibility of targeting Notch-1 in macrophages for therapeutic intervention in AAA is a research direction of interest. Studies have shown that the Notch-1 signaling pathway plays an important role in the occurrence and progression of AAA [5]. Therefore, therapeutic interventions targeting Notch-1 may help regulate the involvement of macrophages in the pathological process, delaying or reversing the progression of AAA. To ensure that such therapeutic interventions have minimal adverse effects on other tissues and systems, further studies are needed. The therapeutic effect can be enhanced by combining Notch-1 inhibitors with other macrophage-targeted therapeutic strategies, such as anti-inflammatory drugs and antioxidants. Increased understanding of the molecular mechanisms of AAA and the role of macrophages in this process will provide more opportunities for clinical translation of therapeutic strategies targeting Notch-1.

In this study, it was first proved that Notch-1 promoted M1 macrophage polarization, and after knockout of Notch-1, M1 macrophage polarization was impaired and M2 polarization was enhanced. M1 macrophages contribute to the occurrence and development of inflammation, secrete inflammatory cytokines, and recruit inflammatory cells and lymphocytes through chemotaxis, thereby further worsening inflammatory responses, and killing and phagocytizing heterologous substances. In contrast, M2 macrophages can promote tissue remodeling, secrete anti-inflammatory cytokines, and negatively regulate the inflammatory response, enhancing the immune escape of tumor cells [11]. It can be inferred that the knockout of Notch-1 will block the development of inflammation and suppress inflammatory cytokines. In addition, SHP2 prevents excess inflammation by directly interacting with MyD88 via its tyrosine 257 [9], and specifically negatively regulates TRIF-mediated gene expression in the TLR signaling pathway partially through inhibiting TBK1-activated signal transduction. To sum up, SHP2 prevents TLR hyperactivation and inflammation. In ROS-mediated oxidative stress, SHP2 is activated and degraded, and it also directly dephosphorylates ANT1 and central molecule controlling mitochondrial permeability transition, thereby preventing collapse of mitochondrial membrane potential and the subsequent release of mitochondrial DNA and ROS [20]. Oxidative stress-SHP2 axis is the key and major regulator for MyD88/TRIF-induced inflammatory responses. In this study, the ApoE^−/−^ chimeric mouse model was established to confirm that Notch-1 regulated the myeloid cell-mediated inflammatory response through the oxidative stress-SHP2 axis in aneurysm formation. Notch-1 disorders are important in the pathogenesis of atherosclerosis, aneurysm and inflammatory response [13, 21]. In this study, it was found that aneurysm tissues had massive macrophage infiltration and a high protein expression of Notch-1 in macrophages. When Notch-1 was knocked out, the pathological damage to the aorta and inflammatory cell infiltration were significantly relieved, abdominal arterial dilation was significantly inhibited and the arterial plaques were reduced. According to the theory of inflammatory response, PRRs on the cell surface and their signal transduction are extremely important in the activation and inflammatory response of myeloid cells. With the deepening of understanding of the important role of inflammatory response in the occurrence and development of aneurysms, TLRs in aneurysm formation have recently become a research hotspot [8]. TLRs include human TLR protein expressed on the surface of cell membrane and in cells [22], which can produce pro-inflammatory cytokines and inflammatory chemokines through ligand recognition, thereby inducing inflammatory responses and regulating innate immunity [23]. Previous studies have verified that TLR-mediated inflammatory responses play an important role in cardiovascular diseases. Edfeldt et al. found that the expression of TLRs significantly rises in coronary atherosclerotic plaques [24] and the targeted knockout of TLR4-encoding genes can reduce aneurysm formation [12–16]. In this study, the TLR-mediated signaling pathway was also highly expressed in aneurysm tissues. After knockout of Notch-1, the activity or phosphorylation levels of corresponding proteins (MyD88, TRIF and NF-κB) were significantly reduced. The intracellular signal transduction of TLRs is achieved mainly in a MyD88-dependent and -independent way. It has been found that MyD88 knockout in hypercholesterolemia mouse models can inhibit the aneurysm formation [12]. Then phagocytosis assay with fluorescent microspheres was designed, and it was found that myeloid-specific knockout of Notch-1 promoted the phagocytic capacity of macrophages by promoting the SHP2 signaling pathway. Other research suggests that ISL exerts its anti-inflammatory effects by inhibiting the activation of the Notch-1/NF-κB and MAPK signaling pathways, which will be the direction of our future research [25–27]. In conclusion, myeloid-specific knockout of Notch-1 can down-regulate the expression of TLR signaling pathway-related proteins, inhibit the oxidative stress level and increase the protein expression of SHP2, thereby relieving the development and formation of aneurysms.
